# AI in UK Medical Education: A Framework for Curriculum Reform

**DOI:** 10.2196/81953

**Published:** 2026-06-12

**Authors:** Aditya Gaur, Joecelyn Kirani Tan, Medha Sridhar Rao, Muhtasim Fuad, Taha Bhatti, Hareesha Rishab Bharadwaj, Khabab Abbasher Hussien Mohamed Ahmed

**Affiliations:** 1 Yeovil District Hospital, Somerset NHS Foundation Trust, Yeovil, United Kingdom; 2 Faculty of Biology, Medicine, and Health, University of Manchester, Oxford Rd, Manchester, M13 9PL, United Kingdom, 44 161 306 6000; 3 School of Medicine, Dentistry and Biomedical Sciences, Queen’s University Belfast, Belfast, United Kingdom; 4 Faculty of Medicine, University of Khartoum, Khartoum, Sudan

**Keywords:** artificial intelligence, AI, medical education, curriculum reform, artificial intelligence literacy, AI literacy, UK health care, medical students, interdisciplinary training, digital health, artificial intelligence ethics, AI ethics, simulation-based learning

## Abstract

Artificial intelligence (AI) is increasingly transforming health care through improvements in diagnosis, predictive analytics, and workflow optimization. However, there remains a significant gap in AI training within UK medical education, leaving future clinicians underprepared for AI-driven health care environments. This viewpoint paper investigated global best practices for AI integration into medical education and proposes a structured framework for embedding AI into the UK medical curriculum. It aimed to assess current attitudes, highlight existing knowledge gaps, and recommend practical implementation strategies. An analysis of international case studies (eg, Stanford University, the University of Toronto, and Chinese University of Hong Kong) was conducted alongside a review of teaching methodologies, stakeholder perspectives, and UK-based surveys to identify core competencies and challenges in AI education. Effective integration strategies include the use of AI-powered simulations, interdisciplinary collaboration, elective modules, and faculty training. Major barriers include lack of AI-literate educators, insufficient ethical training, and limited infrastructure. Knowledge gaps persist among students and faculty in areas such as algorithmic bias, AI ethics, and clinical decision-making. To meet the demands of modern health care, the UK medical curriculum must adopt comprehensive AI training. This includes practical exposure, ethical awareness, and stakeholder engagement. Proactive reform will ensure that graduates are equipped to critically and ethically apply AI tools in clinical practice.

## Introduction

Artificial intelligence (AI) is increasingly embedded in diagnostic, administrative, and clinical decision support systems across health care settings [1,2]. From radiological image analysis to predictive risk stratification and generative documentation tools, AI technologies are reshaping how clinical information is processed and applied. As these systems become more integrated into routine practice, medical education must evolve to ensure that graduates can critically appraise, interpret, and responsibly use AI-enabled tools in patient care [3,4].

Despite AI’s rapid expansion in health care, structured AI education remains limited within UK medical training programs. A recent national survey reported that 88% (432/484) of UK medical students recognized the importance of AI in health care yet only a minority had received formal instruction, highlighting a significant educational gap [5]. This shortfall extends into postgraduate training: in another UK-based survey, 92% (193/210) of trainees felt that AI training in their current curricula was insufficient, and 81% (170/210) of trainees emphasized the necessity of formal AI education [6]. These findings suggest a growing disconnect between technological advancement and educational provision.

Addressing this gap requires more than informal exposure to digital tools. It demands the development of AI literacy, defined in this viewpoint paper as the foundational knowledge, critical appraisal skills, and ethical understanding required to interpret AI outputs, recognize their limitations, communicate their implications to patients, and maintain professional accountability when using AI-supported systems. Therefore, AI literacy encompasses not only technical awareness of concepts such as machine learning and data bias but also the professional judgment necessary to integrate AI safely into clinical reasoning.

**Figure 1.**
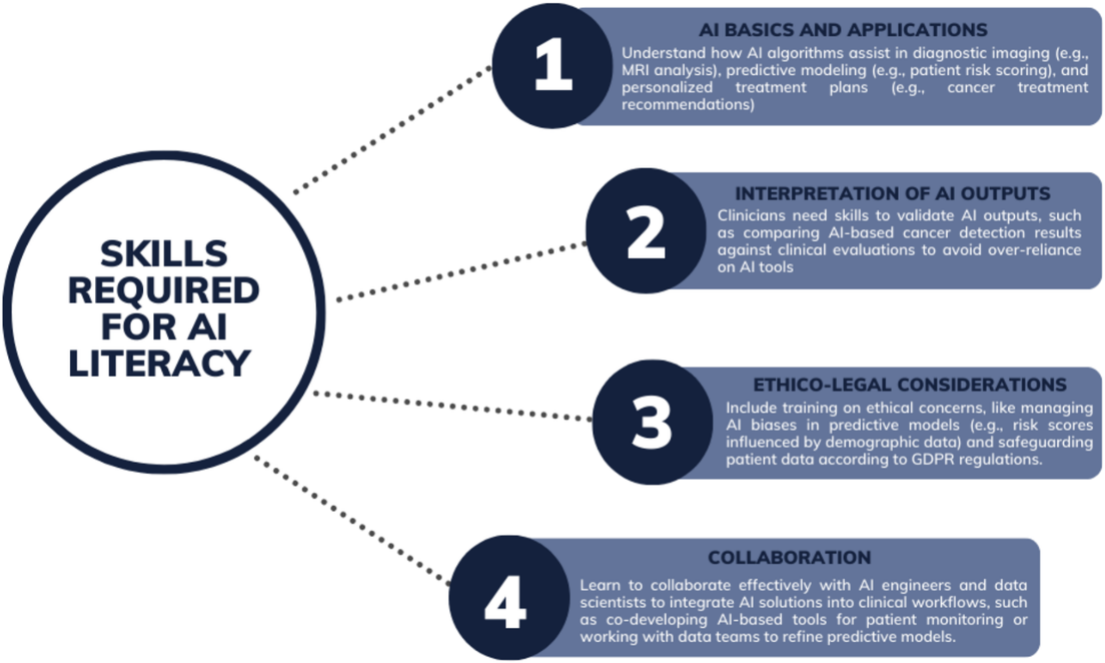
Essential competencies for equipping health care providers for an artificial intelligence (AI)–driven future. GDPR: General Data Protection Regulation; MRI: magnetic resonance imaging.

Research consistently highlights the importance of equipping future clinicians with competencies that enable safe and effective engagement with AI technologies. These competencies, outlined in Figure 1, include foundational technical knowledge, ethical awareness, critical evaluation skills, and applied clinical judgment [7]. However, while numerous publications have discussed AI applications in health care and emerging educational initiatives, few have provided a structured, UK-specific synthesis that integrates international models, regulatory considerations, and competency-based curriculum reform into a unified framework. Existing literature often examines individual teaching interventions or isolated institutional initiatives without systematically analyzing their transferability to centrally regulated systems such as the United Kingdom.

This viewpoint paper addresses that gap by synthesizing current evidence and proposing a structured, context-sensitive framework tailored to the UK medical education system. By evaluating existing educational models, regulatory requirements, and learner perspectives, this viewpoint paper aimed to present a practical road map for developing an AI-literate medical workforce capable of navigating future technological advancements in health care.

## Search Strategy

The review performed in this viewpoint paper was conducted as a structured narrative synthesis aimed at identifying existing approaches to AI integration in undergraduate medical education and evaluating their relevance to the UK context. A defined literature selection process with explicit inclusion and exclusion criteria was applied to enhance methodological transparency and rigor.

Literature searches were conducted across multiple major databases, including PubMed and MEDLINE, Embase, the Cochrane Library, and Scopus. Searches were limited to full-text articles published in English between 2015 and 2025 to reflect contemporary developments in AI technologies. A combination of keywords and Boolean operators was used, including “artificial intelligence,” “AI,” “machine learning,” “deep learning,” “medical education,” “curriculum,” “competency,” and “medical students.” The reference lists of recent review articles were manually screened to identify additional relevant studies.

Studies were included if they (1) examined AI applications within undergraduate or postgraduate medical education; (2) described curricular design, teaching methodology, competency development, or student attitudes; or (3) reported empirical findings, implementation strategies, or institutional case examples. Studies were excluded if they were (1) conference abstracts or proceedings, (2) editorials, (3) case reports unrelated to educational structure, or (4) unpublished. This approach prioritized peer-reviewed evidence describing implemented or evaluated educational initiatives.

International case studies were selected from included studies where institutions demonstrated structured curricular implementation of AI beyond isolated workshops, with clear description of teaching models and institutional support. These examples were synthesized to represent distinct integration models (elective-based, postgraduate specialist and longitudinally embedded approaches) rather than designate a single definitive “best practice.” For the purposes of this viewpoint paper, best practice was defined as initiatives demonstrating interdisciplinary collaboration, sustained curricular integration, ethical governance consideration, and scalability within institutional constraints. UK-based surveys were identified using additional search terms related to UK medical students and AI attitudes, and findings were synthesized narratively to identify recurring gaps in preparedness and training demand. Proposed competency domains were derived through thematic identification of commonly reported educational priorities across included studies and were subsequently mapped conceptually to the outcome framework of the General Medical Council (GMC) to ensure regulatory alignment.

The following sections synthesize findings from the existing literature regarding current gaps, teaching methodologies, barriers, and international models of AI integration in medical education.

## Current Gaps in AI Literacy

For AI to be effectively incorporated into health care education, existing gaps in AI literacy and the scope of AI applications must be addressed. The main gaps stem from a lack of access to AI tools designed for educational use, limited awareness of AI capabilities, fears surrounding AI-generated academic content, inaccuracies in AI outputs, and a shortage of knowledgeable educators. Additionally, there is currently no ethical framework from the GMC or the National Health Service (NHS) guiding AI use in UK medical education. While Health Education England (HEE) developed the Digital, Artificial Intelligence, and Robotics Technologies in Education program [8], it is not widely implemented in UK medical schools. However, the Artificial Intelligence and Digital Healthcare Technologies Capability framework developed by HEE reinforces the national goal of enhancing AI capabilities among health care professionals [8].

Understanding the attitudes of health care professionals and students toward AI is instrumental in enhancing its use in medical practice. Tools such as ChatGPT can aid education by generating questions, flash cards, and writing assistance, enabling students and educators to focus more on learning. However, there is increasing concern about the misuse of AI for academic writing, where students may bypass critical thinking by generating entire essays [9]. This threatens academic integrity, prompting institutions to balance the benefits of AI with safeguards against misuse.

To integrate AI effectively, its role in health care must be aligned with literacy objectives in curricula. Applications such as machine learning for imaging diagnostics, medical administration, drug discovery, and digital twins [10] illustrate AI’s scope.

Despite these knowledge gaps, medical students generally express optimism about AI’s potential to enhance patient outcomes, streamline clinical workflows, and reduce diagnostic errors [11]. Nevertheless, skepticism remains regarding the reliability and transparency of AI systems, with concerns over the risk of overreliance on AI and its potential to perpetuate biases [12].

Faculty perceptions of AI integration appear more mixed than student attitudes. Some faculty members have expressed concern about the potential for AI to replace aspects of traditional medical roles, whereas students are more likely to focus on whether their education adequately prepares them for practice in an AI-enabled health care system [13]. UK survey evidence supports this distinction: while students overwhelmingly recognize the future importance of AI in medicine, they report low levels of confidence in using AI tools in clinical contexts [5].

In contrast, there is currently limited UK-specific empirical evidence examining faculty preparedness or institutional infrastructure gaps. However, international literature suggests that successful integration of AI into medical education depends heavily on educator capability and institutional capacity. A recent global review (January 2026) reported that students across regions consistently favored interactive, practice-based learning over traditional didactic lectures and concluded that meaningful integration requires sustained investment in faculty development and structured curricula addressing both technical and ethical competencies [14].

Similarly, Rani et al [15] found that, although 91% (n=273) of the faculty members surveyed agreed that AI has the potential to enhance medical education, only 12% (n=36) described themselves as “very familiar” with AI technologies. This discrepancy highlights a recurrent international theme: conceptual support for AI integration does not necessarily translate into educator readiness.

Taken together, while UK-specific data on faculty capability and infrastructure remain limited, converging international evidence indicates that AI-literate educators, structured faculty development programs, and accessible technological infrastructure are likely prerequisites for sustainable curriculum reform.

## Teaching Approaches in AI Education

### Overview

Medical education continues to evolve with diverse teaching methodologies aimed at equipping students with the theoretical knowledge and practical skills essential for patient care. As AI becomes increasingly integrated into health care, new approaches are required to ensure that future clinicians are proficient in AI applications. This section explores key teaching methodologies and highlights the existing knowledge gaps and attitudes in preparing medical students for AI-driven clinical environments.

### Hands-On Laboratories

Hands-on laboratories remain a critical component of medical education, providing students with direct experience in anatomical dissection, surgical techniques, and diagnostic procedures. These laboratories enhance learning through experiential engagement, reinforcing conceptual understanding while developing essential clinical skills such as dexterity, coordination, and procedural proficiency [16,17]. However, hands-on training requires substantial resources, including specialized facilities, equipment, and skilled instructors, making it expensive and logistically demanding. Additionally, while these laboratories provide valuable technical training, they do not expose students to the AI-driven tools increasingly used in diagnostics and decision-making [18].

### AI Simulation

AI-powered simulation refers to digitally mediated training environments that use AI algorithms such as machine learning, natural language processing, and adaptive analytics to generate dynamic, responsive clinical scenarios that evolve in real time based on learner input. Unlike traditional static simulations, AI-powered systems can modify patient presentations, diagnostic data, and treatment responses according to a student’s decisions, thereby creating an interactive and personalized learning experience.

AI-powered simulations have become an increasingly valuable tool in medical training, offering students an adaptive environment for clinical decision-making. These systems replicate patient interactions, diagnostic challenges, and treatment planning scenarios, enabling learners to practice clinical reasoning without real-world consequences. AI-driven simulations are particularly beneficial for rare or complex medical cases to which exposure during routine placements may be limited. By providing immediate, data-informed feedback and iterative practice opportunities, such platforms can support the refinement of diagnostic judgment and therapeutic planning skills [19,20].

Despite these advantages, the development and maintenance of high-quality AI simulations remain costly and technically demanding. Infrastructure requirements, data governance considerations, and the need for continual algorithm validation present significant institutional challenges. Moreover, while simulations can enhance learning, they cannot fully replicate the unpredictability, emotional nuance, and contextual complexity of real-world patient care. Therefore, there is a risk that overreliance on simulated environments may limit exposure to authentic clinical uncertainty, reinforcing the need for AI simulation to complement rather than replace supervised clinical experience [21].

### Case-Based Learning

Case-based learning is widely used in medical education to encourage students to apply theoretical knowledge to real or hypothetical patient scenarios. This method promotes critical thinking, diagnostic reasoning, and problem-solving skills, facilitating a deeper understanding of clinical practice [22,23]. In the context of AI education, case studies can be adapted to include AI-assisted diagnostics and treatment recommendations, allowing students to critically assess machine-generated insights. However, the effectiveness of this approach depends on the quality and relevance of the cases presented, as well as the expertise of educators in guiding discussions. Some students may struggle with the complexity of AI-integrated cases, leading to varied learning outcomes [1].

### Online Learning Platforms

With the rise of digital education platforms, online learning has become an increasingly important component of medical training. Online resources, including recorded lectures, interactive modules, and virtual discussions, provide students with flexible, self-paced learning opportunities. This approach is particularly useful for foundational AI education, allowing students to gain exposure to key concepts such as machine learning and natural language processing [11,24]. However, a major limitation of online learning is the lack of direct, hands-on engagement, which is crucial for developing clinical and AI-related competencies. Additionally, online learning requires strong self-discipline and motivation, and it may not fully replace the collaborative and interactive aspects of in-person education [21].

### Knowledge Gaps in AI Competency

As AI technologies become increasingly integrated into health care, it is essential to assess the knowledge gaps and attitudes of medical students and faculty regarding AI. Research suggests that many medical students and faculty possess only a superficial understanding of AI and its applications in clinical practice [25,26]. Fundamental concepts such as machine learning, data analytics, and algorithmic bias remain largely unfamiliar [26].

Beyond technical knowledge, there is also a limited understanding of the ethical and legal implications of AI in health care, including concerns about data privacy, informed consent, and algorithmic transparency [27]. Uncertainty persists regarding AI’s role in clinical decision-making and patient outcomes, particularly in recognizing its strengths and limitations and determining when AI-generated insights should be integrated into patient care [28].

## Barriers to Integration

Despite the potential benefits of AI integration in medical education, several challenges hinder its implementation. One of the primary obstacles is the lack of a structured AI curriculum in most UK medical schools. Medical education traditionally prioritizes biological sciences, human anatomy, physiology, and clinical procedural skills, with limited emphasis on AI-driven domains such as machine learning and data science. Therefore, introducing AI comprehensively into medical training would require curriculum restructuring, protected teaching time, additional resources, and specialized faculty expertise—elements that remain limited within many UK institutions [8].

Ethical considerations pose further challenges, particularly in relation to patient autonomy, data privacy, algorithmic bias, and accountability in AI-assisted decision-making. Maintaining patient confidentiality is a core principle of medical practice, yet AI systems often rely on large-scale data processing that may not fully align with existing ethical and legal standards. Medical students must be equipped to navigate these complexities; however, AI ethics training remains inconsistently embedded in UK medical curricula [29,30]. The integration of structured ethics education into AI training is further constrained by already dense curricula, potentially leaving graduates underprepared to critically evaluate AI’s implications for patient rights and health care equity.

These concerns extend beyond education alone. The 2025 FUTURE-AI consortium, an international consensus involving 117 experts across 50 countries and published in *The BMJ*, emphasized that “trustworthy AI” in health care requires technical robustness, transparency, validation, and traceability throughout the system life cycle [31]. Such standards imply the need for secure data environments, governance frameworks, and systems capable of auditing algorithmic outputs. Many current medical school infrastructures are not configured to support this level of technical integration, underscoring the gap between aspirational AI literacy and the operational requirements of trustworthy clinical AI deployment.

Another barrier is the shortage of AI-proficient faculty. Effective AI education requires instructors with expertise spanning both clinical medicine and AI applications, yet relatively few clinicians possess formal training in data science or machine learning. Recruiting or retraining faculty capable of delivering AI instruction is both time intensive and costly [29,30]. In addition, unfamiliarity with AI concepts may contribute to hesitancy among existing educators, potentially slowing curriculum reform.

These workforce limitations are compounded by financial constraints. The development of AI-focused programs requires sustained investment in computational infrastructure, secure data storage, specialized software, and ongoing system maintenance. For many UK medical schools operating within restricted budgets, allocating long-term funding for such initiatives is challenging [8].

Taken together, these barriers extend beyond short-term logistical concerns. They reflect a broader structural misalignment: effective AI education demands interdisciplinary expertise, governance mechanisms, and technological capacity that traditional medical faculties were not originally designed to support. Without strategic investment, regulatory clarity, and cross-department collaboration, isolated training efforts are unlikely to achieve scalable and sustainable integration.

## International Models of AI Integration in Medical Education

International approaches to AI integration in medical education can be synthesized into three broad curricular models: (1) elective-specialist pathways, (2) postgraduate fellowship expansion, and (3) fully embedded longitudinal curricula. These models differ in scope, scalability, and structural demands, offering distinct implications for centrally regulated systems such as the United Kingdom.

### Elective-Specialist Pathways

Elective-based models introduce AI education through optional modules or symposia that allow interested students to develop specialized competencies. For example, Stanford University incorporates AI through dedicated electives and its Artificial Intelligence in Medicine and Imaging Symposium, which emphasizes experiential learning and practical application in areas such as medical imaging [32].

This model is flexible and relatively low risk as it does not require wholesale curriculum restructuring. However, its scalability is limited. Elective pathways attract students already predisposed toward technology-focused careers, potentially reinforcing inequities in AI literacy across the wider student body [32]. While effective in cultivating future clinician innovators, this model may not ensure baseline AI competency among all graduates.

For systems such as the United Kingdom, where curricular time is tightly regulated, elective integration may represent a feasible entry point. However, its reliance on student self-selection raises concerns regarding universal preparedness.

### Postgraduate Fellowship Expansion

A second model focuses on postgraduate specialization through structured fellowships. At Mayo Clinic, AI-focused fellowships target trainees in areas such as diagnostic imaging and personalized medicine, promoting advanced technical expertise and continuous professional development [32].

This approach strengthens workforce capacity by developing clinician leaders capable of driving AI innovation within health care systems. It is particularly scalable in decentralized systems where institutional autonomy enables specialized program development.

However, as a postgraduate strategy, it does not address foundational AI literacy at the undergraduate level. In centralized systems such as the NHS, fellowship-based expansion could enhance specialist expertise but would not alone ensure system-wide competency among newly qualified physicians.

### Fully Embedded Longitudinal Curricula

The most comprehensive model integrates AI across undergraduate and postgraduate training in a longitudinal manner. At the University of Toronto, AI training is embedded within existing curricula through interdisciplinary collaboration with computer science departments [33]. Similarly, the Chinese University of Hong Kong (CUHK) has implemented AI-powered clinical learning tools, invested in digital laboratory infrastructure, and prioritized faculty development [34].

These models emphasize interdisciplinary learning, ethical reasoning, and sustained exposure to AI applications throughout training. Compared to elective or fellowship approaches, embedded curricula promote equity by ensuring that all students develop baseline AI competency [33,34].

However, this model is structurally demanding. It requires institutional autonomy, protected curriculum time, technological infrastructure, and significant faculty development. In highly centralized and tightly regulated systems, implementation may necessitate coordinated national reform rather than isolated institutional initiatives.

## Structural Constraints and System-Level Implications

While prior literature has primarily documented innovative AI initiatives within individual institutions, less attention has been paid to how such models translate to centrally regulated and publicly funded health care systems. This viewpoint paper moves beyond descriptive comparison to critically evaluate the structural assumptions underlying international models and identify gaps in scalability, equity, and regulatory alignment. By situating AI curriculum reform within the governance and funding realities of UK medical education, this paper advances a system-level framework tailored to a nationally standardized training environment.

International examples illustrate 3 broad models of AI integration: elective-based exposure (eg, Stanford University), postgraduate fellowship pathways (eg, Mayo Clinic), and fully embedded longitudinal curricula (eg, University of Toronto and CUHK) [32-34]. While these differ in intensity, they share enabling conditions: institutional autonomy, protected curricular time, and interdisciplinary faculty capacity. The United Kingdom differs structurally in 2 critical respects.

First, medical education is centrally regulated by the GMC. Therefore, any substantive curricular reform must align with nationally defined graduate outcomes. Unlike more decentralized systems, particularly in the United States, UK institutions cannot independently implement large-scale curriculum redesign without regulatory alignment. This regulatory structure promotes standardization but may slow rapid innovation.

Second, the NHS operates as a publicly funded, centrally coordinated health care system. While this offers opportunities for national alignment, it also constrains financial flexibility. AI-driven educational initiatives require investment in digital infrastructure, simulation platforms, secure data environments, and faculty training. For many UK medical schools operating within fixed budgets and increasing financial pressure, allocating new resources without external funding support may be challenging.

These structural realities suggest that comprehensive, fully embedded AI curricula, as seen in some international institutions, may not be immediately feasible in the United Kingdom without national funding strategies or cross-institutional resource sharing.

## Cost and Implementation Challenges

Beyond high-level structural considerations, practical implementation raises additional challenges.

AI education requires more than curricular time; it demands computational infrastructure, secure data storage, software licensing, and ongoing system maintenance. These recurring costs extend beyond initial development and must be sustained in the long term. In publicly funded institutions facing competing priorities, opportunity costs become a significant consideration: expanding AI content may require the displacement of existing material or additional teaching hours. The NHS Long Term Workforce Plan [35] committed £2.4 billion (US $3.3 billion) to additional training places, but the British Medical Association has noted that funding for simulated learning and AI-ready infrastructure is unevenly distributed, risking a digital divide between older analog hospitals and new digital flagship trusts [36].

Faculty capacity presents an equally significant barrier. AI education requires interdisciplinary expertise spanning data science, ethics, informatics, and clinical medicine—competencies not traditionally embedded within medical faculties. Recruiting new staff or retraining existing faculty entails both financial cost and protected development time. Without clear funding pathways or national coordination, implementation risks are uneven across institutions, potentially exacerbating interschool disparities.

Moreover, variation in curricular structure across UK medical schools introduces logistical complexity. Integration must be sufficiently flexible to accommodate problem-based, system-based, and traditional models of teaching without compromising consistency in learning outcomes.

Taken together, these considerations reinforce the need for incremental and scalable reform rather than immediate wholesale integration.

## Depth, Equity, and Governance

A continuum emerges across international approaches. Elective models maximize flexibility but risk uneven competency distribution. Fellowship pathways cultivate advanced expertise but do not ensure universal literacy. Embedded models promote equity and standardization but require substantial structural investment.

For the United Kingdom, this continuum highlights a central policy tension: balancing depth of expertise with equitable baseline literacy under constrained resources. If AI training remains elective, disparities in confidence and engagement, including gender-based differences reported in European cohorts, may widen [37]. Conversely, universal embedding without adequate faculty preparation risks superficial implementation.

Ethical governance further complicates integration. Institutions must establish clear policies distinguishing legitimate AI-assisted learning from academic misconduct as generative tools become increasingly accessible [38]. Without explicit guidance, ambiguity may undermine professional standards and public trust. Therefore, embedding ethical reasoning and responsible AI use within curricula is not optional but foundational.

The NHS HEE AI Lab provides a relevant example of centrally coordinated workforce development in digital health. Its model demonstrates how national infrastructure can support scalable AI literacy and structured training pathways across the health care system [18]. However, translating such initiatives into undergraduate medical curricula would require careful adaptation, sustainable funding mechanisms, and explicit alignment with the GMC competency framework.

Regulatory expectations further reinforce the urgency of curricular reform. The *Good Medical Practice* update from the GMC emphasizes that physicians should remain professionally accountable for all clinical decisions, including those informed by AI systems [39]. This position is reflected in professional attitudes: a 2024 survey conducted by the Alan Turing Institute supported by the GMC found that, although many physicians report using AI tools, 99% (814/822) would prioritize their own clinical judgment over an AI recommendation in cases of conflict [40].

Despite growing enthusiasm for AI integration, several gaps remain insufficiently addressed in the existing literature. First, few studies examine long-term sustainability or cost-effectiveness beyond initial implementation. Second, there is limited evaluation of competency outcomes, particularly whether exposure translates into measurable clinical decision-making proficiency. Third, the regulatory implications of AI competency standards in nationally governed systems remain underexplored. Finally, most initiatives focus on technologically advanced institutions, leaving questions about equity of access across diverse educational settings. These unresolved issues are particularly salient in the UK context and underscore the need for a coordinated, evaluative, and economically conscious integration strategy.

## A Phased Framework for UK Integration

### Overview

In light of these feasibility, cost, regulatory, and workforce considerations, a phased approach is most appropriate for the UK context. Reform should begin with a comprehensive needs assessment to establish baseline attitudes, knowledge gaps, institutional readiness, and available faculty capacity. Survey evidence from UK medical students demonstrates strong recognition (432/484, 88%) of AI’s future importance alongside low levels of confidence in applying AI tools, underscoring the need for structured intervention rather than informal exposure [5]. As of 2025, a total of 88% (916/1041) of UK undergraduate students report using generative AI tools (up from 53% in 2024), yet only 36% (375/1041) report receiving formal institutional training [41]. Therefore, initial assessments must evaluate not only learner demand but also financial viability, digital infrastructure capacity, and alignment with existing program outcomes defined by the GMC.

### Mapping AI Competencies to GMC Outcomes

To ensure regulatory coherence, AI competencies should be explicitly mapped to the GMC’s outcomes for graduates domains: (1) professional values and behaviors: understanding ethical AI use, data governance, algorithmic bias, and responsible integration into patient care; (2) professional skills: critically appraising AI-generated outputs, communicating AI-informed decisions to patients, and maintaining clinical accountability when AI recommendations conflict with professional judgment; and (3) professional knowledge: foundational literacy in machine learning principles, data limitations, validation processes, and real-world clinical applications.

Rather than positioning AI as a discrete subject, embedding competencies within these existing domains reduces curricular displacement while reinforcing that AI literacy is integral to safe, modern clinical practice.

### Curriculum Design and Module Structure

Within a pilot module or integrated teaching block, illustrative learning objectives may include (1) describing the basic principles underlying machine learning and clinical decision support systems, (2) critically evaluating the strengths and limitations of AI tools in imaging or diagnostic contexts, (3) identifying ethical risks associated with algorithmic bias and data misuse, and (4) demonstrating appropriate professional judgment when AI outputs conflict with clinical reasoning.

A feasible structure could involve a blended model combining (1) introductory lectures (foundational concepts), (2) case-based seminars integrating AI into clinical scenarios, (3) simulation-based exercises using AI-assisted diagnostic tools, and (4) interdisciplinary workshops codelivered with data science faculty.

Such integration mirrors approaches observed in international embedded longitudinal models, including those at the University of Toronto and CUHK [33,34], while adapting them to the structural constraints of UK programs.

### Assessment Strategies

Assessment should align with existing formats to maintain feasibility. Potential approaches include (1) incorporating AI critical appraisal questions into written examinations, (2) objective structured clinical examination stations requiring interpretation of AI-assisted outputs, (3) reflective essays on ethical AI dilemmas, and (4) workplace-based assessments evaluating appropriate AI integration in simulated scenarios.

Embedding AI evaluation within established assessment structures avoids the need for parallel examination systems while reinforcing accountability and patient safety principles.

### Faculty Development and Infrastructure Requirements

Faculty development is central to sustainable implementation. Institutions may adopt a tiered approach: (1) foundational AI literacy workshops for all teaching staff, (2) advanced training for designated AI curriculum leads, and (3) cross-department collaboration with computer science or informatics departments.

Minimum infrastructure requirements include secure digital platforms for simulation exercises, access to validated AI demonstration tools, and data governance protocols consistent with NHS standards. National coordination through NHS-linked digital education initiatives may reduce duplication and promote equitable access across institutions.

### Progressive Integration

Early pilot programs such as student-selected components or elective modules should serve as evaluation platforms to quantify resource requirements, faculty workload implications, and cost sustainability. As institutional capacity develops, competencies can be progressively embedded within core modules such as radiology, diagnostics, ethics, and clinical decision-making.

Continuous evaluation should extend beyond assessment modalities to clearly defined outcome indicators. Institutions should establish baseline AI literacy and faculty readiness metrics prior to implementation followed by structured pre- and postintervention comparisons. Suggested indicators include validated AI literacy or digital health competency scales (where available), performance in AI-integrated objective structured clinical examination stations, case-based ethics assessments examining bias recognition, learner confidence surveys, and faculty self-reported preparedness following development programs. A phased evaluation timeline—baseline (before implementation), pilot year assessment, and longitudinal review at 2 to 3 years—would enable quantification of competency attainment, cost-effectiveness, and sustainability. Defining these metrics explicitly ensures that AI integration generates evaluable evidence rather than remaining a conceptual reform initiative. The phased framework for AI integration in UK undergraduate medical education is summarized in Table 1.

**Table 1.** Phased framework for artificial intelligence (AI) integration in UK undergraduate medical education.

Phase	Key components	Practical strategies	Evaluation metrics	References
Needs assessment and institutional readiness	Establish baseline learner knowledge, attitudes, faculty capacity, and infrastructure	Survey AI literacy and confidence among students Assess faculty readiness and expertise Review digital infrastructure and financial feasibility Align with GMC^a^ outcomes for graduates	Baseline AI literacy scores Student confidence surveys Faculty preparedness metrics Infrastructure audit	[5,41]
Competency mapping to GMC outcomes	Establish baseline learner knowledge, attitudes, faculty capacity, and infrastructure	Map AI competencies to professional values and behaviors (ethics, bias, and governance), professional skills (AI appraisal, communication, and accountability), and professional knowledge (ML^b^ principles, validation, and limitations)	Explicit curriculum mapping Regulatory alignment review	GMC outcomes for graduates
Curriculum design and delivery	Embed AI into existing modules rather than as a stand-alone subject	Introductory lectures (foundations) Case-based seminars AI-assisted simulation exercises Interdisciplinary workshops with data science faculty	Module participation rates Learner feedback Objective knowledge assessments	[33,34]
Assessment integration	Align AI evaluation with existing assessment structures	AI critical appraisal questions in written exams AI-integrated OSCE^c^ stations Reflective essays on ethics and bias Workplace-based assessments	Performance in AI-specific exam items OSCE scoring outcomes Ethics case analysis performance	[7]
Faculty development and infrastructure	Develop sustainable teaching capacity	Tiered AI literacy workshops Advanced training for curriculum leads Collaboration with informatics departments Secure simulation platforms and validated tools	Faculty training completion rates Faculty confidence surveys Infrastructure compliance with NHS^d^ standards	[18]
Progressive integration and longitudinal evaluation	Scale from pilot modules to core curriculum	Begin with electives or student-selected components Expand into radiology, diagnostics, ethics, and clinical reasoning Establish phased evaluation timeline (baseline, year 1 pilot, and 2- or 3-year review)	Pretest-posttest AI literacy comparisons AI-integrated OSCE performance trends Learner confidence shifts Faculty readiness improvements Cost-effectiveness analysis	[5,41]

a GMC: General Medical Council.

b ML: machine learning.

c OSCE: objective structured clinical examination.

d NHS: National Health Service.

## Conclusions

AI is no longer a speculative addition to health care but an operational reality shaping diagnosis, decision-making, and system design. However, UK medical education has not evolved at a comparable pace. This viewpoint paper demonstrates that, while international institutions have piloted diverse models of AI integration, sustainable reform within the United Kingdom requires a regulatory-aligned, economically conscious, and evaluative approach. Developing AI literacy must extend beyond technical familiarity to encompass ethical reasoning, critical appraisal, and professional accountability in AI-supported clinical environments. A phased, competency-mapped framework grounded in GMC standards, supported by faculty development, and reinforced through measurable outcome indicators offers a pragmatic pathway forward. Without structured integration, disparities in preparedness may widen, and graduates risk entering practice underequipped for an AI-enabled NHS. Therefore, proactive, coordinated curriculum reform is not simply an educational innovation but a professional imperative to safeguard patient care, uphold public trust, and ensure that future physicians remain critically engaged leaders in an increasingly intelligent health care system.

## Abbreviations

AI: artificial intelligence
CUHK: Chinese University of Hong Kong
GMC: General Medical Council
HEE: Health Education England
NHS: National Health Service
